# Population dynamics of three lizard species from the genus *Sceloporus*: short‐term changes in demographic parameters

**DOI:** 10.1111/1749-4877.12396

**Published:** 2019-11-25

**Authors:** Selene VARGAS‐GARCÍA, Víctor ARGAEZ, Israel SOLANO‐ZAVALETA, J. Jaime ZÚÑIGA‐VEGA

**Affiliations:** ^1^ Graduate Program in Biological Sciences National Autonomous University of Mexico, University City Mexico City México; ^2^ Department of Ecology and Natural Resources, Faculty of Sciences National Autonomous University of Mexico, University City Mexico City México

**Keywords:** abundance, recruitment, *Sceloporus* lizards, survival, temporary emigration

## Abstract

Most demographic studies focus on numerical changes that occur within populations across years. However, typically studies at an interannual scale do not provide information on the particular times of the year (particular months or seasons) when rates of survival, recruitment, or migration increase or decrease due to physiological, behavioral or ecological processes. These monthly or seasonal changes in demographic parameters may lead to substantial variations in population abundance. In this study, we collected capture–mark–recapture data on 3 species of lizards of the genus *Sceloporus* (*Sceloporus torquatus, Sceloporus grammicus* and *Sceloporus megalepidurus*) found in ecologically similar habitats to examine potential changes in demographic rates among 3 different climatic seasons: rainy, cold‐dry and warm‐dry seasons. We tested different hypotheses about the effect of these seasons on survival, recruitment of new adults, and temporary emigration. We found that during the season with severe thermal constraints, the cold‐dry season, survival of *S. torquatus* decreased markedly. We also detected a considerable increase in the recruitment rate of *S. grammicus* during the rainy season, when these lizards are establishing territories and finding mates. In contrast, we found no evidence of intra‐annual changes in the rate of temporary emigration. In addition, we calculated abundance and population growth rates for each species and for each season. Our study represents a significant contribution to the understanding of intra‐annual demographic variation in lizards.

### INTRODUCTION

Most demographic studies aim to understand interannual variation in population parameters such as survival and abundance (Oro *et al*. 2010; LaManna *et al*. 2012; Sandvik *et al*. 2012). Even though yearly estimates are important, interannual studies do not provide information on the specific times of the year during which critical demographic events occur (Grosbois *et al*. 2008). For example, several studies on birds have documented years in which both survival and abundance decrease dramatically (e.g. Sillett *et al*. 2000; Wright *et al.* 2009; Paxton *et al.* 2014; Ancona *et al.* 2017). These studies attempt to identify the causes of these decreases through correlations between their demographic estimates and the average climatic conditions of their studied years. However, these annual censuses do not provide information on the times of the year (e.g. seasons) when critical processes like emigration or reproduction occur, which may be responsible for the observed decreases in survival and abundance. In mammals, several long-term studies have documented years with drastic reductions in population density (e.g. Owen-Smith *et al*. 2005; Wang *et al.* 2009; Havemann *et al.* 2016). At this scale, it is not possible to distinguish whether mortality increases during specific periods, such as the reproductive season or the winter. Therefore, studies based on monthly or seasonal censuses are required to determine the times of the year when critical biological or ecological processes lead to changes in the vital rates (Grosbois *et al*. 2008; Levy *et al*. 2015).

In lizards, there are studies that analyze interannual variation in vital rates, such as fecundity or survival, and relate these variations to some biotic or abiotic factor that could be affecting these demographic parameters (Le Galliard *et al*. 2010; Pastro *et al*. 2013; Wolf *et al*. 2014; Ujvari *et al*. 2015). For instance, survival in 4 species of the genus *Ctenotus* increases in years with more precipitation (Read *et al.* 2012). In contrast, the abundance of the lizard *Uta stansburiana* decreases in years of increased precipitation (Flesch *et al*. 2017). With this type of study, we cannot know at what specific time of the year the effect of precipitation (whether positive or negative) on survival and abundance occurs.

Although less common, there are also some studies on lizards based on censuses conducted on a shorter time scale (i.e. monthly or seasonal) that document within-year variation in demographic parameters. These short-term censuses show that temperature, precipitation and food availability during particular months, as well as the reproductive season, can notably affect the vital rates of these organisms (Niewiarowski *et al*. 2004; Endriss *et al*. 2007; Ramírez-Bautista *et al*. 2016). Females of *Xenosaurus grandis* have higher mortality during the months that coincide with the late gestation and early postpartum periods (Zúñiga-Vega 2011). The abundance of juveniles of 2 species of the genus *Ctenophorus* increases considerably during the fall when precipitation from the previous summer was relatively high, apparently because abundant rain favors the survival and growth of the young (Dickman *et al*. 1999). Studies at the intra-annual scale on lizards are still needed to understand how biological and ecological processes that vary throughout the year (i.e. climatic and reproductive seasons) affect the population parameters of these organisms.

In this study, we examined different demographic parameters in 3 species of lizards of the genus *Sceloporus* inhabiting central Mexico: *Sceloporus torquatus* Wiegmann, 1828, *Sceloporus grammicus* Wiegmann, 1828 and *Sceloporus megalepidurus* Smith, 1934. We implemented capture–mark–recapture procedures, sampling approximately every 3 months. Our main objective was to know during which season of the year changes in survival, recruitment of new adults, temporary emigration and population abundance occur. In addition, we tested 3 hypotheses that attempt to explain the causes of seasonal variation in these demographic parameters.

Our first hypothesis suggests a decrease in survival in all 3 species during the cold-dry season because lizards are ectothermic organisms whose metabolism and behavior depend on environmental temperature (Angilletta *et al*. 2010; Le Galliard *et al*. 2010; McKay & Phillips 2012). Low temperatures constrain metabolic activity, which, in turn, may cause increased mortality (Ashton 2001; Chamaillé-Jammes *et al*. 2006). As a consequence of reduced survival, population abundance should also decrease during this season.

Our second hypothesis focused on recruitment of new reproductive individuals. We expected an increase in this parameter just before the breeding season, because adults initiate the search and establishment of territories and potential mates (Calsbeek & Sinervo 2002; Cooper & Vitt 2002; Smith & Lemos-Espinal 2005). In addition, before the breeding season, new individuals that have just reached sexual maturity also join the search for mates (Ortega-León *et al*. 2007). In all 3 species, breeding begins towards the end of the rainy season (Godínez-Cano 1985; Feria-Ortiz *et al*. 2001; Ramírez-Bautista *et al*. 2012). Therefore, we expected the highest recruitment of adults to occur during this season. Because of the increase in recruitment of new adults, we expected an increase in population abundance during the rainy season.

Finally, our third hypothesis focused on the rate of temporary emigration, which refers to the probability that an individual is temporarily outside the study area

(i.e. not available for recapture) during a particular season, with subsequent return to the study area (Pollock *et al*. 1990; Kendall *et al*. 1997). The rate of temporary emigration is, therefore, determined by the movement of individuals across space. Individuals that carry out their activities within a small area have a low probability of being outside the study site during any particular census, whereas individuals that move across longer distances (i.e. that have larger activity areas) have a higher probability of being outside the study area during any particular census. We predicted an increase in the movement of individuals and, in consequence, increased temporary emigration, during the warm-dry season for 2 reasons. First, during most of this season, males of the 3 study species are not breeding (Godínez-Cano 1985; Feria-Ortiz *et al*. 2001; Ramírez-Bautista *et al*. 2012) and, therefore, are not strictly restricted to their territories. Second, during the warm-dry season, food (i.e. invertebrates) is less abundant (Ramírez-Bautista & González-Romero 2002) and, therefore, both males and females must move more to find prey.

### MATERIAL AND METHODS

#### Study areas

We studied 3 lizard species of the genus *Sceloporus* at ecologically similar sites. For *S. torquatus*, we worked in a botanical garden within an ecological reserve (Reserva Ecológica del Pedregal de San Ángel, REPSA) located in southern Mexico City (19.31826°N, −99.19431°W). The REPSA has an elevation of 2300 m a.s.l. The main vegetation type is xerophytic scrub, dominated by the bush *Pittocaulon praecox* (Feria-Ortiz *et al*. 2001; Rojo & Rodríguez 2002; Argaez *et al*. 2018). The study area for *S. grammicus* was located in the vicinity of Nopala de Villagrán, in the state of Hidalgo (20.26834°N, −99.64171°W) at an elevation of 2400 m a.s.l. This is a semi-arid region with xerophytic scrub (Flores-Villela & Canseco-Márquez 2007; Gómez Mendoza 2007; Roth-Monzón *et al*. 2018). The study area for *S. megalepidurus* was the area around Lake Alchichica (19.40568°N, −97.40240°W), located in the state of Puebla at an altitude of 2436 m a.s.l. (Hernández Márquez 2016). The predominant vegetation is xerophytic scrub, with plants belonging to the genera *Agave*, *Opuntia*, *Dasylirion* and *Nolina* (González-Ruiz 1991; Alcocer *et al*. 2000; Rzedowski 2016). In all 3 study sites, the rainy season occurs from June through October and the dry season from November through May.

#### Study species

*Sceloporus torquatus*, *S. grammicus* and *S. megalepidurus* are all viviparous, insectivorous lizard species inhabiting central Mexico (Sites *et al*. 1992; Ramírez-Bautista & González-Romero 2002; Leyte-Manrique & Ramírez-Bautista 2010). *S. torquatus* is a saxicolous lizard (Smith 1936; Duellman 1961). Males and females of this species reach sexual maturity at a minimum snout–vent length (SVL) of 73 mm (Feria-Ortiz *et al*. 2001). Matings occur in the fall (October–November). Vitellogenesis begins at the end of the summer (August–September) and ovulation occurs at the end of November and the beginning of December (Fig. 1). Females are

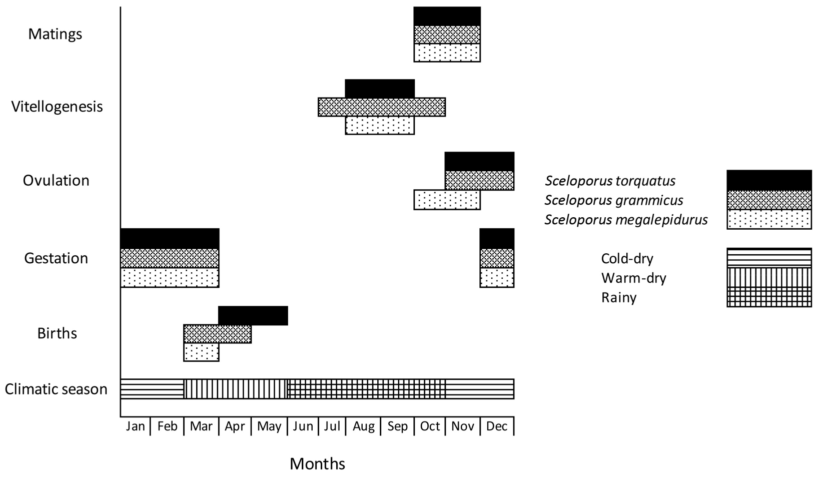

**Figure 1** Duration of different phases of the reproductive cycles of 3 lizard species of the genus *Sceloporus*. We also show the duration of 3 climatic seasons: rainy, cold-dry, and warm-dry.

pregnant over the winter (December–March) and young are born the following spring (April–May) (Feria-Ortiz *et al*. 2001; Ramírez-Bautista & González-Romero 2002). This species exhibits sexual dimorphism; adult males are larger than females and have large colorful (blue and black) ventral patches. Females also have colorful patches on their bellies, but these are smaller and less intense than in males (Martínez-Méndez & Méndez-de la Cruz 2007).

*Sceloporus grammicus* is a predominantly arboreal species (Ramírez-Bautista *et al*. 2009). Size at maturity is 42.5 mm SVL in males and 40.7 mm SVL in females (Pérez-Mendoza & Zúñiga-Vega 2014). Matings occur in the fall (October–November) (Pérez-Mendoza *et al*. 2014). Vitellogenesis begins in the summer (July–October) and ovulation occurs between November and December (Fig. 1). Females are pregnant during the winter and young are born in early spring (Guillette & Casas-Andreu 1980; Ortega & Barbault 1984; Ramírez-Bautista *et al*. 2004, 2005, 2012; Jiménez-Cruz *et al*. 2005). This species is also sexually dimorphic; males have blue, orange or yellow patches on their throats and black and blue ventral patches, whereas females lack these patches and can present slightly orange coloration on their bellies (Ramírez-Bautista *et al*. 2012).

*Sceloporus megalepidurus* is a species that uses a diversity of plants (yuccas, agaves and cacti) as microhabitat (Smith 1939; Sánchez-Herrera 1980). Males reach sexual maturity at 45 mm SVL, whereas females mature at 37 mm SVL (Godínez-Cano 1985; González-Ruiz 1991). Matings also occur in the fall (October–November), vitellogenesis occurs during the rainy season (August–September), ovulation occurs in October and November, females are pregnant over the winter, and young are born in March (Fig. 1; Sánchez-Herrera 1980; González-Ruiz 1991). There is no evident sexual dimorphism in this species; both sexes lack color patches on the belly or throat.

### Field methods

In the 3 study areas, we delimited a plot of approximately 1 ha in which we captured adult males and females by hand or noose. In the REPSA botanical garden, we sampled *S. torquatus* 9 times (an average of once every 2 months) between August 2015 and March 2017. In Nopala de Villagrán, we sampled *S. grammicus* 7 times (on average every 3 months) between April 2016 and October 2017. In Lake Alchichica, we sampled *S. megalepidurus* 7 times (on average every 2.5 months) between February 2016 and June 2017. Each sampling period lasted between 5 and 8 consecutive days. Each captured lizard was measured (± 0.01 mm SVL), weighed (± 0.1 g) and individually marked with a unique combination of small scars on the ventral surface of the limbs using a medical cautery pen (Ekner *et al*. 2011; Argaez *et al*. 2018). We determined the sex of each lizard by the presence (males) or absence (females) of enlarged post-anal scales (Mayhew 1963; Doughty *et al*. 1994). During each subsequent visit to each site, we recorded the number of recaptured individuals and marked lizards that had not been previously captured.

### Mark–recapture analyses

We analyzed the capture–mark–recapture data using the program MARK, which uses maximum likelihood procedures to estimate several demographic parameters (White & Burnham 1999). In all analyses we focused exclusively on adult individuals (i.e. lizards whose SVL was larger than that reported in the literature as minimum length at sexual maturity). We considered that the demographic parameters of these 3 lizard species could vary drastically among 3 climatic seasons that clearly differ in temperature and precipitation. The rainy season begins in June and ends in October. We divided the dry season into 2 periods: the cold-dry season, from November to February, and the warm-dry season, from March to May.

In all 3 sites, our total sampling period spanned approximately 1.5 years. This means that, for 1 or 2 seasons (depending on the particular site) we collected data from 2 different years (e.g. for *S. grammicus* we collected data from 2 different warm-dry seasons and 2 different rainy seasons). When we attempted to estimate demographic rates separately for all these seasons (i.e. when estimating the effect of year), the resulting parameter estimates were not precise enough (i.e. had large standard errors) to detect differences among seasons or to derive reliable inferences. Therefore, we pooled data from the same season (e.g. the 2 warm-dry seasons were pooled together) and omitted the effect of year. The resulting parameter estimates were quite accurate and, thus, here we report all our demographic rates from the analyses that omitted the effect of year. In the supplementary materials we show the full results that accounted for differences between years, which were in general consistent with the results from the pooled data.

We used Pradel models for open populations (Pradel 1996) to estimate the apparent survival rate (φ), recruitment rate (*f*), recapture probability (*p*) and population growth rate (λ). Specifically, the recruitment rate (*f*) refers to the number of reproductive individuals that enter the population for each adult individual already present in the population. The Pradel models allow the estimation of λ as the sum of φ and *f* (Franklin 2001). To implement these Pradel models, we combined data from consecutive daily visits in such a way that, pooled together, these multiple consecutive visits represented a single occasion for each sampling period.

We constructed alternative models representing different hypotheses about variation in φ, *f* and *p*. We considered a null or constant model (.), variation among seasons (*e*), between sexes (*s*), an additive effect of sex and season ($s + e$), and a sex by season interaction ($s \times e$). In addition, for recapture probability, we evaluated the effect of average temperature (averaged across all the consecutive daily visits, *temp*), an additive effect of temperature and sex ($s + temp$), and a sex by temperature interaction ($s \times temp$). Considering all possible combinations of these sources of variation resulted in a total of 200 models per species.

We selected the best model based on the Akaike information criterion adjusted for small sample sizes (AICc). The smallest AICc value indicates the model that provides the best fit to the data. Models that differ by less than 2 AICc units (ΔAICc < 2) from the best model are also strongly supported by the data (Akaike 1973; Burnham & Anderson 2002). In addition, we calculated the AICc weight (*w*) for each model, which represents a relative measure of the strength of evidence in favor of each model. Based on these AICc weights, we generated weighted averages (across all models) of φ, *f*, *p* and λ for each combination of sex and season (Burnham & Anderson 2002).

To estimate population abundance (*N*) and rate of temporary emigration (γ), we used the robust design of Pollock *et al.* (1990), in which the sampling scheme consists of primary periods separated by long time intervals (2 or 3 months in our case), which, in turn, are composed of secondary sampling periods separated by shorter time intervals (consecutive days in our case). During these secondary periods, the robust design assumes that the population is demographically closed, allowing the estimation of population abundance. We tested this assumption by comparing the fit of a model in which we fixed φ = 1 and *f* = 0 during the secondary periods (no deaths or recruitment, as expected in a closed population) to the fit of a model in which we allowed the estimation of φ and *f* during these secondary periods (thereby allowing for some deaths and recruitment, as expected in an open population). In all 3 species, the model assuming population closure provided a better fit than the model that represented an open population (ΔAICc = 4 in all 3 cases). In between the primary sampling periods, the population is open, so additions (births and immigration) and subtractions (deaths and emigration) can occur, because they are separated by longer time intervals (Kendall 1999). Therefore, the robust design also allows the estimation of survival (φ) and temporary emigration (γ), as well as recapture probability (*p*) (Pollock *et al*. 1990).

The rate of temporary emigration (γ) is defined as the probability that an individual is temporarily outside the study area, and, therefore, not available for recapture during a particular primary sampling period. According to Kendall *et al*. (1995, 1997), this parameter can be expressed in 2 forms (γ′ and γ″). The parameter γ′ represents the probability that an individual is not available for capture during a primary sampling occasion (*i*) given that the animal was not present in the study area during the previous primary sampling occasion ($i - 1$). On the other hand, γ″ represents the probability that an individual is not available for capture during a given primary sampling occasion (*i*) given that the animal was present in the study area during the previous primary sampling occasion ($i - 1$). We did not distinguish between these 2 forms of temporary emigration because when estimating both parameters their standard errors were too large. We therefore decided to set γ′ equal to γ″ in all our models.

Using the robust design, we constructed different models to test biological hypotheses with respect to variation in φ, γ and *p*. Population abundance (*N*) was calculated as a derived parameter. As in Pradel models, we considered a null model (.), variation among seasons (*e*) and between sexes (*s*), an additive effect of sex and season ($s + e$), and a sex by season interaction ($s \times e$). In addition, for recapture probability, we considered the effect of daily temperature (in this case, the mean temperature of each day of sampling, *daily temp*), as well as additive and interactive effects of daily temperature and sex ($s$ + *daily temp* and $s \times$ *daily temp*, respectively).

Unfortunately, our data were insufficient to appropriately estimate seasonal variation in temporary emigration for 2 of the 3 study species: *S. grammicus* and *S. megalepidurus* (i.e. the standard errors estimated for this parameter for different seasons were too large). Therefore, for these 2 species we only considered a null model and the effect of sex on γ, resulting in a total of 80 models. For *S. torquatus* we did estimate seasonal variation in γ, yielding a total of 200 models for this species.

Once again, we selected the best model based on AICc and calculated weighted averages of φ, γ, *p* and *N* for each combination of sex and season, considering the AICc weight (*w*) of each model (Burnham & Anderson 2002). However, model-averaged estimates of φ and *p* for each sex and season derived from the robust design models were very similar to these parameters estimated from the Pradel models. Therefore, for simplicity, we only report model-averaged survival and recapture rates derived from the Pradel models. For *S. grammicus* and *S. megalepidurus* we also report the effect of mean temperature per secondary sampling period (i.e. mean temperature of each sampling day, *daily temp*) on *p*, given that the best-fitting robust design models included this effect. We report all demographic parameters on a monthly scale.

### RESULTS

For *S. torquatus*, we marked a total of 497 adult individuals, of which 297 were females and 200 were males. Of these, we recaptured 141 individuals at least once, of which 90 were females and 51 were males. For *S. grammicus*, we marked a total of 737 adult individuals, of which 422 were females and 315 were males. Of these, 215 individuals were recaptured at least once, of which 118 were females and 97 were males. For *S. megalepidurus*, we marked a total of 689 adult individuals, of which 362 were females and 327 were males. Of these, 173 individuals were recaptured at least once, of which 93 were females and 80 were males.

#### Pradel models

*Sceloporus torquatus*

The monthly survival rate of *S. torquatus* was notably high (>0.8 in all cases) and clearly varied among seasons: the 3 best-supported models (ΔAICc < 2) included the effect of season (Table 1). Although the effect of sex was included in 2 of the best models, the third of these models with strong support did not include this factor (Table 1). This means that considering differences be-

**Table 1** Best-fitting Pradel models that examine variation in rates of survival (φ), recapture (*p*) and recruitment (*f*) of 3 lizard species of the genus *Sceloporus*

| Species | Model | AICc[†] | ΔAICc[‡] | *w*[§] | Deviance | *K*[¶] |
|---|---|---|---|---|---|---|
| *Sceloporus torquatus* | φ (*s* + *e*) *p* (*s* × *e*) *f* (*s* × *e*) | 3424.22 | 0 | 0.34 | 344.19 | 16 |
| | φ (*s* × *e*) *p* (*s* × *e*) *f* (*s* × *e*) | 3425.26 | 1.05 | 0.20 | 341.03 | 18 |
| | φ (*e*) *p* (*s* × *e*) *f* (*e*) | 3425.83 | 1.61 | 0.15 | 354.14 | 12 |
| *Sceloporus grammicus* | φ (*s* + *e*) *p* (*s* × *e*) *f* (*s* × *e*) | 4413.69 | 0 | 0.28 | 184.63 | 16 |
| | φ (*s* × *e*) *p* (*s* × *e*) *f* (*s* + *e*) | 4414.68 | 0.99 | 0.17 | 185.63 | 16 |
| | φ (*s* × *e*) *p* (*s* × *e*) *f* (*e*) | 4415.55 | 1.87 | 0.11 | 188.56 | 15 |
| *Sceloporus megalepidurus* | φ (*e*) *p* (.) *f* (*s* + *e*) | 3834.09 | 0 | 0.08 | 131.44 | 8 |
| | φ (*e*) *p* (*temp*) *f* (*s* + *e*) | 3834.13 | 0.04 | 0.08 | 129.44 | 9 |
| | φ (.) *p* (*e*) *f* (*s* + *e*) | 3834.15 | 0.06 | 0.08 | 131.51 | 8 |
| | φ (*e*) *p* (*temp*) *f* (*s* × *e*) | 3834.41 | 0.32 | 0.07 | 125.63 | 11 |
| | φ (*e*) *p* (*s*) *f* (*s* + *e*) | 3834.97 | 0.88 | 0.05 | 130.29 | 9 |
| | φ (*s* + *e*) *p* (*temp*) *f* (*s* + *e*) | 3835.19 | 1.10 | 0.05 | 128.45 | 10 |
| | φ (.) *p* (*s* + *e*) *f* (*s* + *e*) | 3835.20 | 1.11 | 0.05 | 130.51 | 9 |
| | φ (.) *p* (*e*) *f* (*s* × *e*) | 3836.02 | 1.94 | 0.03 | 129.29 | 10 |

The demographic rates may vary as a function of sex (*s*), season (*e*), the additive effect of sex and season (*s* + *e*), and the interaction between sex and season (*s* × *e*). We also considered intercept-only (null) models (.). In addition, for recapture probability (*p*), we evaluated the effects of average temperature (*temp*), the additive effect of average temperature and sex (*temp* + *s*), and the interaction between average temperature and sex (*temp* × *s*). [†]The fit of each model was evaluated using the Akaike information criterion adjusted for small sample sizes (AICc). [‡]ΔAICc indicates the difference in the AICc value of each model with respect to the top model. We only show models with ΔAICc < 2. [§]Akaike weights, which indicate the relative support for each model in the data. [¶]Number of parameters in each model.

tween males and females in φ did not substantially improve model fit compared to the model that only included the effect of season. Survival of both sexes decreased in the cold-dry season and was substantially higher during the rainy season (Fig. 2a).

Season also clearly affected the recruitment rate: the 3 best models included this factor (Table 1). In addition, in this case, the effect of sex was not entirely evident. Recruitment in males and females was practically zero during the cold-dry season and relatively high (between 0.14 and 0.20) during both the rainy and warm-dry seasons (Fig. 2b). We could not adequately estimate the recruitment rate of females during the rainy season.

As a result of decreased survival and recruitment during the cold-dry season, the rate of population growth for both sexes was statistically less than 1 during this season (Fig. 2c). In contrast, λ was greater than 1 for both sexes during the warm-dry season and for males during the rainy season, whereas λ for females was statistically equal to 1 during the rainy season (Fig. 2c). When we accounted for differences between years, we obtained similar results: φ, *f* and λ decreased substantially during the 2 different cold-dry seasons (Fig. S1a–c).

The 3 best-supported models indicated an effect of the interaction between sex and season on the recapture probability of *S. torquatus* (Table 1). During the rainy and cold-dry seasons, we did not detect a difference between sexes in *p*, whereas during the warm-dry season, the recapture probability for females was higher than for males (Fig. 2d).

*Sceloporus grammicus*

The monthly survival rate of *S. grammicus* was also notably high (>0.8 in all cases). The 3 best-supported models included the effects of season and sex (Table 1). The top model suggested an additive effect of these 2 factors and adding the interaction term did not substantially improve the model fit (Table 1). The weighted averages of φ showed a slight increase in female survival during the cold-dry season and a slight decrease in survival of both sexes during the rainy season (Fig. 3a).

According to the 3 best-supported models, recruitment of *S. grammicus* clearly varied among seasons without a clear effect of sex (Table 1). This parameter was equal to zero in males and quite close to zero in females during the cold-dry season, with intermediate values during the warm-dry season (0.05 and 0.08 for males and females, respectively) and higher values during the rainy season (0.18 and 0.13 for males and females, respectively) (Fig. 3b).

The rate of population growth was statistically less than 1 only for males during the cold-dry season (Fig. 3c), the season during which recruitment of this sex was equal to zero (Fig. 3b). When accounting for differences between years, we also observed slight decreases in φ during the 2 rainy seasons (Fig. S2a), as well as the lowest *f* and $\lambda < 1$ during the cold-dry season (Fig. S2b-c).

The 3 best-supported models indicated an effect of the interaction between sex and season on the recapture probability of *S. grammicus* (Table 1). During the warm-dry season, we did not detect differences between sexes in *p*. During the rainy season, recapture probability was higher for males, whereas during the cold-dry season recapture probability was higher for females (Fig. 3d).

*Sceloporus megalepidurus*

As in the other 2 species, the monthly survival rate of *S. megalepidurus* was notably high (>0.85 in all cases). Given that the model with constant survival (null model) had strong support in the data (ΔAICc = 0.06 with respect to the top model), including the effects of sex or season did not improve model fit (Table 1). We thus found little evidence of differences between sexes or among seasons in φ (Fig. 4a).

In contrast, the effects of sex and season were clearly evident in the recruitment rate of *S. megalepidurus* (Table 1). Recruitment was quite close to zero during the rainy and cold-dry seasons, with an evident increase in this parameter during the warm-dry season (Fig. 4b). During this latter season, males had a higher recruitment rate than females (0.32 and 0.19, respectively; Fig. 4b).

The rate of population growth for both sexes was less than 1 during the cold-dry season (Fig. 4c), when *f* was practically zero (Fig. 4b). In contrast, during the warm-dry season, λ of both sexes was statistically greater than 1 (Fig. 4c), which was consistent with the substantial increase in *f* of both males and females during this season. When we accounted for differences between years, we also obtained similar results: lack of variation in φ between sexes or among seasons (Fig. S3a), increased *f* during the 2 warm-dry seasons (Fig. S3b), λ < 1 during the cold-dry season, and λ > 1 during the 2 warm-dry seasons (Fig. S3c).

The model with constant *p* (null model) ranked first in this species (Table 1). Therefore, we found no evidence of differences between sexes or among seasons in the recapture probability of *S. megalepidurus* (Fig. 4d).

### Robust design models

*Sceloporus torquatus*

The model considering a constant emigration rate (null model) ranked first in this species (Table 2). The model indicating a difference between sexes in γ ranked second and also had strong support in the data (ΔAICc = 0.19). However, estimating the additional parameter that represented the effect of sex did not substantially improve the model fit and, hence, evidence of intersexual differences in temporary emigration was actually weak. The effect of season had weak support in the data (ΔAICc = 9.34 and $w = 0.005$ for the first model that included differences among seasons in γ). During all seasons, the proportion of individuals that moved outside the study area was similar between sexes, around 0.4 and 0.5 (Fig. 5a).

In no season did we detect statistical differences between males and females in population abundance. Abundance of females was almost identical during the rainy and warm-dry seasons (around 212 individuals), whereas for males the estimate of $N$ varied between 136 and 177 individuals (Fig. 5b). During the cold-dry sea-

**Table 2** Best-fitting robust design models that examine variation in rates of survival (φ), recapture ($p$) and temporary emigration (γ) of 3 lizard species of the genus *Sceloporus*

| Species | Model | AICc[†] | ΔAICc[‡] | $w$[§] | Deviance | $K$[¶] |
|---|---|---|---|---|---|---|
| *Sceloporus torquatus* | φ ($s + e$) γ (.) $p$ ($s \times e$) | 5250.78 | 0 | 0.52 | 4645.77 | 11 |
| | φ ($s + e$) γ ($s$) $p$ ($s \times e$) | 5250.97 | 0.19 | 0.47 | 4643.90 | 12 |
| *Sceloporus grammicus* | φ ($s \times e$) γ (.) $p$ (*daily temp* × $s$) | 6533.26 | 0 | 0.23 | 6541.90 | 11 |
| | φ ($s \times e$) γ (.) $p$ (*daily temp*) | 6533.36 | 0.10 | 0.22 | 6546.07 | 9 |
| | φ ($s \times e$) γ (.) $p$ (*daily temp* + $s$) | 6535.06 | 1.80 | 0.09 | 6545.73 | 10 |
| *Sceloporus megalepidurus* | φ ($s \times e$) γ (.) $p$ (*daily temp* × $s$) | 5096.81 | 0 | 0.15 | 4652.31 | 11 |
| | φ ($e$) γ (.) $p$ (*daily temp* × $s$) | 5097.36 | 0.55 | 0.11 | 4658.99 | 8 |
| | φ (.) γ (.) $p$ (*daily temp* × $s$) | 5097.68 | 0.87 | 0.09 | 4663.37 | 6 |
| | φ ($s \times e$) γ ($s$) $p$ (*daily temp* × $s$) | 5098.66 | 1.85 | 0.06 | 4652.11 | 12 |

The demographic rates may vary as a function of sex ($s$), season ($e$), the additive effect of sex and season ($s + e$) and the interaction between sex and season ($s \times e$). We also considered intercept-only (null) models (.). In addition, for recapture probability ($p$), we evaluated the effects of daily temperature (*daily temp*), the additive effect of daily temperature and sex (*daily temp* + $s$) and the interaction between daily temperature and sex (*daily temp* × $s$). [†]The fit of each model was evaluated using the Akaike information criterion adjusted for small sample sizes (AICc). [‡]ΔAICc indicates the difference in the AICc value of each model with respect to the top model. We only show models with ΔAICc < 2. [§]Akaike weights, which indicate the relative support for each model in the data. [¶]Number of parameters in each model.

son, *N* decreased notably for both sexes, with approximately 104 females and 85 males (Fig. 5b). Our estimates of population abundance that accounted for differences between years also indicated relatively low numbers of individuals during the 2 cold-dry seasons (Fig. S1d).

*Sceloporus grammicus*

In this species, the 3 best-supported models included a constant emigration rate (Table 2). For both sexes and all seasons, γ was approximately 0.20 (Fig. 6a). In contrast, we did detect differences between sexes in *N* during the cold-dry and warm-dry seasons. Abundance of males was lower (between 125 and 161 individuals) than abundance of females (between 319 and 324 individuals) during both seasons (Fig. 6b). During the rainy season we did not find evidence of differences between sexes in this parameter (our estimates were 299 males and 337 females; Fig. 6b). Estimates of *N* that accounted for differences between years also revealed that males were less abundant than females in 3 of the 5 seasons that we visited this population (Fig. S2d).

In the case of recapture probability estimated using the robust design, the 3 best-supported models included an effect of daily temperature (Table 2). Adding an effect of sex did not notably improve the fit of these models and, therefore, *p* was similar between sexes. Apparently, *p* of both males and females decreased slightly with increasing daily temperature (Fig. 6c).

*Sceloporus megalepidurus*

As in the other 2 species, we found strong support for a constant emigration rate (Table 2). Even though the model that ranked fourth included an effect of sex on γ and had considerable support in the data (ΔAICc = 1.85), the addition of the parameter that represented the effect of sex did not improve the model fit. Thus, we found little evidence of differences between males and females in temporary emigration. For all seasons, γ was estimated as 0.15 for males and 0.17 for females (Fig. 7a).

Abundance of both sexes notably decreased during the cold-dry season (around 175 males and 301 females), compared to the rainy (around 486 males and 546 females) and warm-dry (around 391 males and 578 females) seasons (Fig. 7b). Similar to *S. grammicus*, the abundance of *S. megalepidurus* females was higher than that of males during the cold-dry and warm-dry seasons (Fig. 7b), although these differences between sexes were not as drastic as in *S. grammicus* (Fig. 6b). Estimates of *N* from the models that accounted for differences between years revealed similar patterns: reductions in population abundance during the 2 cold-dry seasons and greater abundance of females also during the 2 cold-dry seasons as well as during 1 warm-dry season (Fig. S3d).

The 4 best-supported models included an effect of the

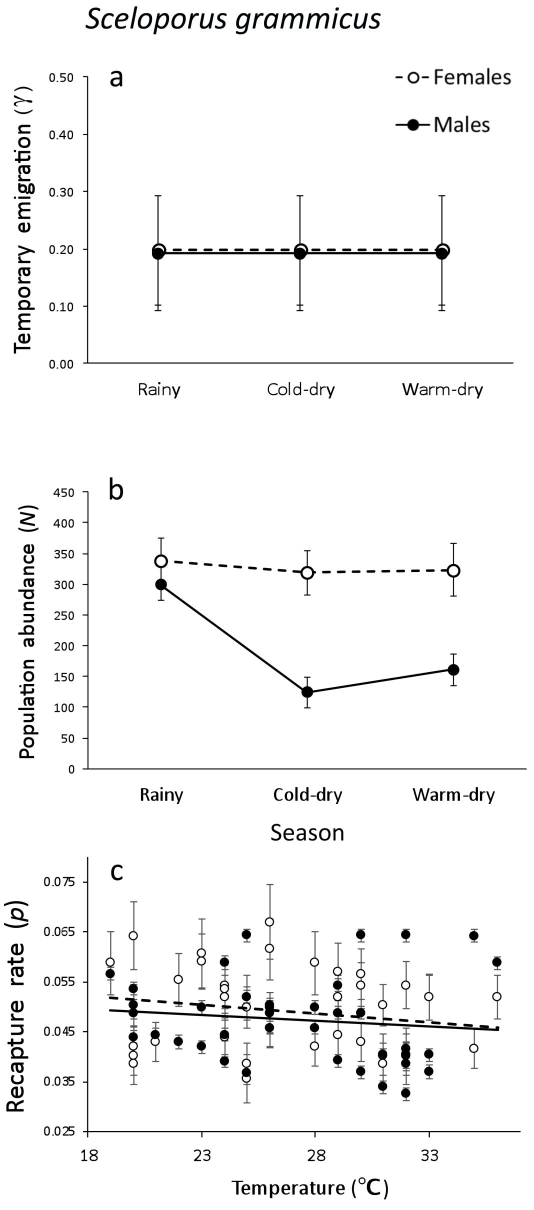

**Figure 6** Model-averaged estimates of temporary emigration (a) and population abundance (b) for males and females of *Sceloporus grammicus.* We also show the estimated effects of daily temperature on recapture rates (c). Error bars indicate 1 standard error.

interaction between daily temperature and sex on *p* (Table 2). The estimate of *p* for males decreased with increasing daily temperature, whereas *p* for females was not affected by this environmental variable (Fig. 7c).

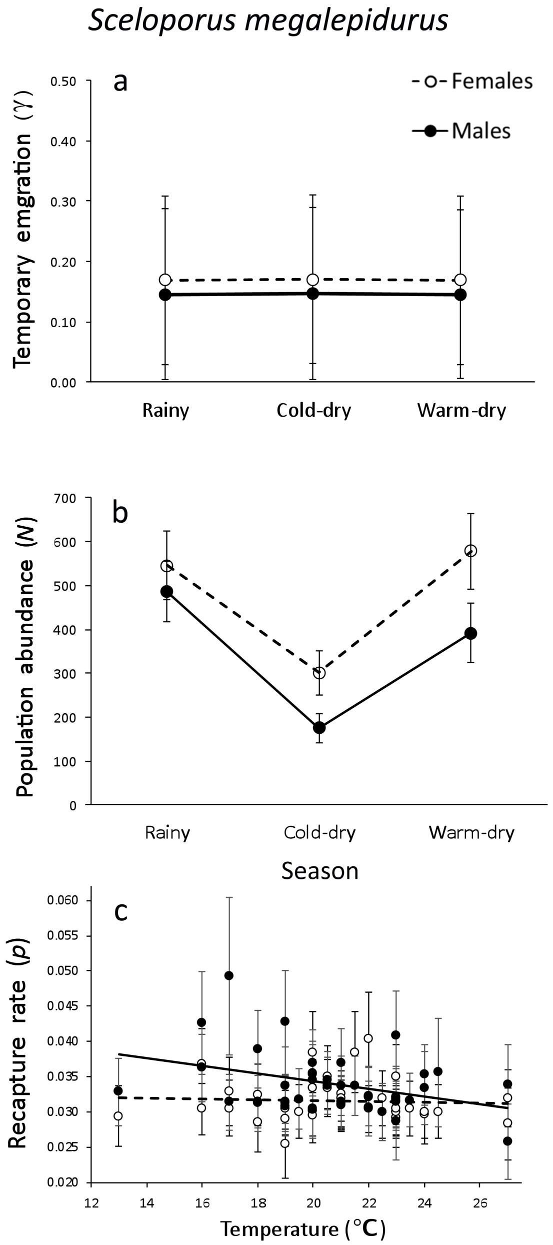

**Figure 7** Model-averaged estimates of temporary emigration (a) and population abundance (b) for males and females of *Sceloporus megalepidurus*. We also show the estimated effects of daily temperature on recapture rates (c). Error bars indicate 1 standard error.

## DISCUSSION

The analysis of demographic rates at an intra-annual scale allowed us to observe the time of the year when the most drastic changes in survival, recruitment and abundance occurred in our 3 study species. In addition, we report for the first time in the genus *Sceloporus* estimates of temporary emigration from the study area. Our findings revealed an interesting general pattern: in all 3 species the number of individuals and the rate of population growth were lowest during the cold-dry season. This pattern has 2 important implications. First, harsh environmental conditions and critical reproductive processes (e.g. gestation; Fig. 1) that occur during these cold months appear to cause increased mortality and/or restricted recruitment of new adults into the population. Second, interannual changes in population numbers of these and other species may be explained by the occurrence of severe winters. The observed negative effects of cold-dry months on vital rates may certainly occur in other ectothermic vertebrates, such as other reptiles and many amphibians (Ashton 2001; Anholt *et al*. 2003). In endothermic vertebrates (birds and mammals), whose metabolism does not depend as strongly on environmental temperature, the negative demographic consequences of the cold-dry season might not be as drastic, although this hypothesis that predicts differences between ectothermic and endothermic vertebrates deserves further exploration.

In contrast, the rainy and warm-dry seasons had, in general, positive effects on the population numbers of our 3 study species. Positive population growth during these seasons may have resulted from the combination of favorable climatic conditions and less demanding phases of the annual reproductive cycle (males are not reproductive during a large part of these 2 seasons; Fig. 1). This is consistent with the positive population growth that occurs during the spring and summer in several other taxa that inhabit environments with stronger seasonality (such as regions located at higher latitudes), caused by warmer temperatures and abundant food resources during these seasons (Merritt *et al*. 2001; Cornulier *et al*. 2013). Thus, particular years of high population abundance that have been observed in numerous animal species (e.g. Owen-Smith *et al*. 2005; Campbell *et al*. 2015) may have been the result of remarkably good conditions during the warm and/or rainy seasons. In addition, our results also demonstrate interesting differences among the 3 lizard species that we studied, which we discuss in the context of the hypotheses that we proposed for this study.

**Hypothesis 1.** *Survival decreases during the cold-dry season.*

This hypothesis was confirmed only in 1 of our 3 study species: *S. torquatus*. Survival of both sexes decreased during the cold-dry season. Temperature has a critical influence on all physiological processes of ectothermic organisms (Huey *et al*. 2009; Kearney 2013; Abram *et al*. 2017). In lizards, there is a diversity of studies on the impact of temperature on processes such as digestion, disease resistance, gestation and activity (Angilletta *et al.* 2002; Kearney & Porter 2004; Le Galliard *et al*. 2010; McKay & Phillips 2012). Therefore, drastic reductions in environmental temperature could lead to severe alterations of vital functions, such as metabolism and the immune system, which could, in turn, lead to increased mortality (Angilletta *et al*. 2004, 2010; Chamaillé-Jammes *et al*. 2006). Only 22% of juveniles of the lizard *Sceloporus undulatus* survive the winter (Jones & Ballinger 1987). In the snake *Crotalus viridis*, smaller individuals have lower survival rates during the winter due to physiological costs of hibernation (Ashton 2001). In the lizard *Lacerta vivipara*, higher temperatures during spring and summer are directly related to increased survival (Chamaillé-Jammes *et al.* 2006).

We did not find support for the hypothesis that survival decreases during the cold-dry season in *S. grammicus* or *S. megalepidurus*. In contrast, in *S. grammicus* we observed that females had a slightly higher survival rate in the cold-dry season, and in *S. megalepidurus* we found no differences in survival among seasons. These differences from what we observed in *S. torquatus* can be tentatively explained in 2 ways. First, decreases in temperature in the microhabitats occupied by *S. torquatus* may be more intense than those experienced by *S. grammicus* and *S. megalepidurus*. The study area for *S. torquatus* is a botanical garden with greater tree cover, which may lead to increased thermal constraints (i.e. fewer refuges and perch sites that receive direct sunlight). In contrast, the study sites for *S. grammicus* and *S. megalepidurus* are xerophytic scrubs, where vegetation is much more open. Second, increased survival in female *S. grammicus* during the cold-dry season could be due to differences in behavior compared to the other 2 species. Even though females from all 3 species are pregnant during the winter (Fig. 1) and during gestation many species of lizards have lower mobility and forage less frequently (Weiss 2001; Sinervo *et al*. 2010), female *S. grammicus* could have the lowest activity levels compared to the other 2 species, making them notably less conspicuous to predators.

An alternative explanation for the lower survival during the cold-dry season that we detected in *S. torquatus* is that both males and females may experience reproductive costs. Gestation and matings take place during this season (Fig. 1) and these reproductive processes are energetically demanding (Harshman & Zera 2007). Large investments in embryo development and in securing and defending mates and territories can entail a higher risk of mortality (Marler *et al.* 1995; Zúñiga-Vega 2011). Intriguingly, we did not detect evidence of these trade-offs between these reproductive processes and survival of *S. grammicus* and *S. megalepidurus*, even though in all 3 species gestation and matings occur during the cold-dry season.

**Hypothesis 2.** *Recruitment increases during the rainy season.*

Given that matings in all 3 species begin toward the end of the rainy season, we proposed the hypothesis that just before matings (i.e. during the rainy season), new adult individuals should recruit into the population. Both males and females must search for potential mates and establish territories during the rainy season, which would lead to the arrival of new adult individuals to the study area. We found support for this hypothesis only in *S. grammicus*, in which the recruitment rate of both sexes was highest during the rainy season.

In contrast, the recruitment rate for adults of *S. megalepidurus* was notably lower during the rainy and cold-dry seasons. During the warm-dry season, in contrast, approximately 6 months before matings, recruitment in this species was relatively high: 0.19 females for each female present in the population and 0.32 males for each male present in the population. Therefore, in this species recruitment is apparently not associated with the search for mates, but rather could be the result of searching for sites with higher food availability during the driest and warmest time of the year, when the abundance of potential prey (i.e. invertebrates) is notably low (Ramírez-Bautista *et al*. 2016). This hypothesis was not supported in *S. torquatus* either. Recruitment of adults in this species was relatively high during both the rainy season and the warm-dry season. Thus, this demographic process is not strictly associated with the pre-mating season in this species either.

**Hypothesis 3.** *Temporary emigration increases during the warm-dry season.*

The rate of temporary emigration is associated with the movement of individuals throughout space. We proposed the hypothesis that the 3 species of lizards would move more during the non-reproductive season, when they are not restricted to territories, which is the warmest and driest season of the year. This season also coincides with the months during which food availability is lowest, when individuals should have to move the most in order to find prey. The prediction derived from this hypothesis was that the rate of temporary emigration out of the study area would be highest during the warm-dry season. Unfortunately, we were only able to test this hypothesis using the data from *S. torquatus* (due to convergence problems when season was incorporated as a factor affecting the rate of temporary emigration of the other 2 species). In this species, we did not find evidence of variation due to season or sex. We must notice that the values estimated for this parameter were quite high: on each sampling occasion between 40% and 50% of marked individuals were outside the study area. This is evidence that these lizards move considerable distances throughout the year, regardless of the phase of the reproductive cycle.

Our study is the second to estimate this demographic parameter for lizard species. The gecko *Nephrurus stellatus* in Australia had a temporary emigration rate of 0.093 (i.e. on each sampling occasion 9.3% of marked individuals were temporarily outside the study area; Smith *et al*. 2012). This estimate of temporary emigration is considerably lower than what we observed in *S. torquatus*, suggesting that this latter species moves around relatively large areas.

### Abundance and population growth rate

In general, our results were consistent in terms of the expected effects of seasonal variation in recruitment and survival on population abundance. In *S. torquatus*, reductions in survival and recruitment of both sexes during the cold-dry season led to decreased abundance and population growth rate during this season. In *S. grammicus*, the abundance of males decreased notably during the cold-dry and warm-dry seasons, apparently due to drastic reductions in recruitment during these 2 seasons. In fact, the population growth rate of males indicated a substantial decrease in the number of adult males during the cold-dry season, when recruitment was practically zero. In contrast, the increase in female survival during the cold-dry season compensated for the decrease in female recruitment during this season, leading to relatively stable abundance and population growth rate throughout the year for females. At the same time, this generated a female-biased sex ratio in *S. grammicus* during the dry periods of the year. Other studies on lizards have also found that females are more abundant than males, for example in the lizard *Sceloporus scalaris* (Ortega-Rubio *et al*. 2000) and in the dwarf chameleon *Bradypodion pumilum* (Katz *et al*. 2013).

In *S. megalepidurus,* both abundance and population growth rate were lowest during the cold-dry season, when recruitment of both sexes was practically zero. During the warm-dry season, individuals of both sexes were recruited into the population, generating positive population growth rates during this season. In this species we also detected greater abundance of females during both dry seasons compared to the abundance of males. This female-biased sex ratio could have interesting consequences in terms of the intensity of sexual selection (McCoy *et al*. 2004; Bock *et al*. 2010). If there are relatively few males, then male–male competition for potential mates should be relaxed, which could reduce the magnitude of sexual size dimorphism (i.e. relatively smaller males; Berns 2013). Consistent with this hypothesis, the most intense sexual size dimorphism occurs in *S. torquatus*, which is the species in which we did not find differences in abundance between males and females and in which males are notably larger than females (Ramírez-Bautista & González-Romero 2002). In *S. grammicus* there is moderate sexual size dimorphism (Jiménez-Cruz *et al*. 2005) and in *S. megalepidurus* there is no sexual size dimorphism (González-Ruiz 1991).

Our estimates of population abundance indicate that our 3 study species are locally abundant. If we take into account that the 3 study sites had an average area of 1 ha, the lowest population density we observed was 85 lizards/ha in *S. torquatus* during the cold-dry season, and the highest was 578 lizards/ha in *S. megalepidurus* during the warm-dry season. These densities are relatively high compared to densities of other species from the same genus, such as *S. undulatus* (63.5 lizards/ha; Buckley *et al*. 2008), *S. scalaris* (48 lizards/ha; Ortega-Rubio *et al*. 2000) and *S. woodi* (5 lizards/ha; McCoy *et al*. 2004).

### Caveats and future directions

We recognize that our total sampling period lasted on average 1.5 years. This implies that we had limited temporal coverage. Even though we obtained similar patterns of demographic variation when we pooled data from 2 different years and when we accounted for potential differences between years (see our supplementary materials), longer monitoring of these populations may reveal greater interannual variation. Hence, the seasonal patterns that we detected here should be interpreted with caution because previous long-term studies have demonstrated particular years with unusual conditions during either the favorable (warm or rainy) or the restricting (cold or dry) season, which in turn had substantial effects on interannual demographic variation (e.g. DelGiudice *et al.* 2002; Jónsson *et al.* 2013). In other words, the data that we collected here may have come from an unusually harsh cold-dry season or from unusually benign warm and rainy seasons, which do not represent the average environmental conditions in our study sites.

In addition, we focused our study exclusively on adult individuals because we recaptured very few juveniles, which precluded us from accurately estimating demographic parameters for these immature individuals. Juveniles are an important and sometimes large component of all animal populations and, thus, must be considered when making inferences with respect to population dynamics. Thus, we propose the following informed hypothesis about seasonal variation in the vital rates of juveniles of our 3 study species. First, juvenile survival should be lower than adult survival, with an evident decrease also during the cold-dry season, such as has been observed in other lizard species (Civantos *et al*. 1999; Siqueira & Rocha 2008). Second, recruitment of juveniles should be highest right after parturitions take place; that is, at the end of the warm-dry season and at the beginning of the rainy season (Fig. 1; Vogel 1984). Third, juveniles should have higher rates of temporary emigration than adults because in other lizard species (and in general in many vertebrates) juveniles are less philopatric and have higher dispersal rates than adults (Warner & Shine 2008; Vercken *et al*. 2012). Testing these hypotheses requires more effective ways to recapture juvenile individuals in natural conditions.

### ACKNOWLEDGMENTS

Funding was provided by Dirección General de Asuntos del Personal Académico-Universidad Nacional Autónoma de México through project PAPIIT IN203516. We thank the following people for field assistance: Abril Villagrán, Marco Aguilar, Sergio Porto and Saúl Osorio. Mariana Hernández-Apolinar, Hibraim Pérez-Mendoza and Rodrigo Macip-Ríos provided helpful academic advice. Raquel Ortiz-Fernández, Aline Vargas-García and Gonzalo Ramírez-Cruz helped in the preparation of figures. Fieldwork was conducted with permission from Secretaría de Medio Ambiente y Recursos Naturales-México (SGPA/DGVS/11338/15). We also thank José Manuel Tivo Guzmán, Othón Cervantes Sánchez and the Comisión Nacional del Agua (CONAGUA) for providing climatic information. The first author is grateful to the Posgrado en Ciencias Biológicas, Universidad Nacional Autónoma de México for continued academic support and to the Consejo Nacional de Ciencia y Tecnología (CONACyT) for the scholarship received (779026).

### SUPPLEMENTARY MATERIALS

**Cite this article as:**

## Cite this article as:

Vargas‐García S, Argaez V, Solano‐Zavaleta I, Zúñiga‐Vega JJ (2019). Population dynamics of three lizard species from the genus *Sceloporus*: short‐term changes in demographic parameters. *Integrative Zoology* **14**, 542–60.

## Supporting information

Additional supporting information may be found in the online version of this article at the publisher's website.
**Figure S1** Model‐averaged estimates of survival (a), recruitment (b), population growth (c) and population abundance (d) for males and females of *Sceloporus torquatus*. These demographic estimates account for potential differences between years. Error bars indicate 1 standard error.
**Figure S2** Model‐averaged estimates of survival (a), recruitment (b), population growth (c) and population abundance (d) for males and females of *Sceloporus grammicus*. These demographic estimates account for potential differences between years. Error bars indicate 1 standard error.
**Figure S3** Model‐averaged estimates of survival (a), recruitment (b), population growth (c) and population abundance (d) for males and females of *Sceloporus megalepidurus*. These demographic estimates account for potential differences between years. Error bars indicate 1 standard error.

Supporting Information

## References

[inz212396-bib-0001] Abram PK , Boivin G , Moiroux J , Brodeur J (2017). Behavioural effects of temperature on ectothermic animals: Unifying thermal physiology and behavioural plasticity. Biological Reviews 92, 1859–76. 28980433 10.1111/brv.12312

[inz212396-bib-0002] Akaike H (1973). Information theory and an extension of the maximum likelihood principle. In: Petrov BN, F Czáki, eds. Second International Symposium on Information Theory. Akademiai Kiadó, Budapest, Hungary, pp. 267–81.

[inz212396-bib-0003] Alcocer J , Lugo A , Escobar E , Sánchez MR , Vilaclara G (2000). Water column stratification and its implications in the tropical warm monomictic Lake Alchichica, Puebla, Mexico. Verhandlungen Internationalis Vereinigung Limnologiae 27, 3166–9.

[inz212396-bib-0004] Ancona S , Dénes FV ., Krüger O , Székely T , Beissinger SR (2017). Estimating adult sex ratios in nature. Philosophical Transactions of the Royal Society B: Biological Sciences 372, 20160313. 10.1098/rstb.2016.0313 PMC5540855 28760756

[inz212396-bib-0005] Angilletta MJ , Niewiarowski PH , Navas CA (2002). The evolution of thermal physiology in ectotherms. Journal of Thermal Biology 27, 249–68.

[inz212396-bib-0006] Angilletta MJ , Niewiarowski PH , Dunham AE , Leaché AD , Porter WP (2004). Bergmann's clines in ectotherms: illustrating a life‐history perspective with Sceloporine lizards. The American Naturalist 164, E168–83. 10.1086/425222 29641924

[inz212396-bib-0007] Angilletta MJ , Cooper BS , Schuler MS , Boyles JG (2010). The evolution of thermal physiology in endotherms. Frontiers in Bioscience E2, 861–81. 10.2741/e148 20515760

[inz212396-bib-0008] Anholt BR , Hotz H , Gaston-Denis G , Semlitsch RD (2003). Overwinter survival of *Rana lessonae* and its hemiclonal associate Rana esculenta. Ecology 84, 391–7.

[inz212396-bib-0009] Argaez V , Solano-Zavaleta I , Zúñiga-Vega JJ (2018). Another potential cost of tail autotomy: tail loss may result in high ectoparasite loads in *Sceloporus* lizards. Amphibia-Reptilia 39, 191–202.

[inz212396-bib-0010] Ashton KG (2001). Body size variation among mainland populations of the Western rattlesnake (Crotalus viridis). Evolution 55, 2523–33. 11831667 10.1111/j.0014-3820.2001.tb00766.x

[inz212396-bib-0011] Berns CM (2013). The evolution of sexual dimorphism: Understanding mechanisms of sexual shape differences. In: Moriyama H, ed. Sexual Dimorphism, 1st edn. InTech, Croatia, pp. 1–16.

[inz212396-bib-0012] Bock BC , Zapata AM , Páez VP (2010). Survivorship rates of adult *Anolis mariarum* (Squamata: Polychrotidae) in two populations with differing mean and asymptotic body sizes. Papéis Avulsos de Zoologia 50, 43–50.

[inz212396-bib-0013] Buckley LB , Rodda GH , Jetz W (2008). Thermal and energetic constraints on ectotherm abundance: a global test using lizards. Ecology 89, 48–55. 18376546 10.1890/07-0845.1

[inz212396-bib-0014] Burnham KP , Anderson DR (2002). Model Selection and Multimodel Inference: A Practical Information‐Theoretic Approach, 2nd edn. Springer-Verlag, New York, USA.

[inz212396-bib-0015] Calsbeek R , Sinervo B (2002). Uncoupling direct and indirect components of female choice in the wild. Proceedings of the National Academy of Sciences 99, 14897–902. 10.1073/pnas.242645199 PMC137516 12417752

[inz212396-bib-0016] Campbell GS , Thomas L , Whitaker K , Douglas AB , Calambokidis J , Hildebrand JA (2015). Inter-annual and seasonal trends in cetacean distribution, density and abundance off southern California. Deep Sea Research Part II: Topical Studies in Oceanography 112, 143–57.

[inz212396-bib-0017] Chamaillé-Jammes S , Massot M , Aragón P , Clobert J (2006). Global warming and positive fitness response in mountain populations of common lizards Lacerta vivipara. Global Change Biology 12, 392–402.

[inz212396-bib-0018] Civantos E , Salvador A , Veiga JP (1999). Body size and microhabitat affect winter survival of hatchling *Psammodromus algirus* lizards. Copeia 1999, 1112–7.

[inz212396-bib-0019] Cooper W , Vitt L (2002). Increased predation risk while mate guarding as a cost of reproduction for male Broad‐headed skinks (Eumeces laticeps). Acta Ethologica 5, 19–23.

[inz212396-bib-0020] Cornulier T , Yoccoz NG , Bretagnolle V *et al* (2013). Europe-wide dampening of population cycles in keystone herbivores. Science 340, 63–6. 23559246 10.1126/science.1228992

[inz212396-bib-0021] DelGiudice GD , Riggs MR , Joly P , Pan W (2002). Winter severity, survival, and cause‐specific mortality of female white‐tailed deer in north‐central Minnesota. Journal of Wildlife Management 66, 698–717.

[inz212396-bib-0022] Dickman CR , Letnic M , Mahon PS (1999). Population dynamics of two species of dragon lizards in arid Australia: the effects of rainfall. Oecologia 119, 357–66. 28307758 10.1007/s004420050796

[inz212396-bib-0023] Doughty P , Sinervo B , Burghardt GM (1994). Sex-biased dispersal in a polygynous lizard, Uta stansburiana. Animal Behaviour 47, 227–9.

[inz212396-bib-0024] Duellman WE (1961). The amphibians and reptiles of Michoacán, México. University of Kansas Publications, Museum of Natural History 15, 1–148.

[inz212396-bib-0025] Ekner A , Sajkowska Z , Dudek K , Tryjanowski P (2011). Medical cautery units as a permanent and non‐invasive method of marking lizards. Acta Herpetologica 6, 229–36.

[inz212396-bib-0026] Endriss DA , Hellgren EC , Fox SF , Moody RW (2007). Demography of an urban population of the Texas horned lizard (*Phrynosoma cornutum*) in Central Oklahoma. Herpetologica 63, 320–31.

[inz212396-bib-0027] Feria-Ortiz M , Nieto-Montes de Oca A , Salgado Ugarte IH (2001). Diet and reproductive biology of the viviparous lizard *Sceloporus torquatus torquatus* (Squamata: Phrynosomatidae). Journal of Herpetology 35, 104–12.

[inz212396-bib-0028] Flesch AD , Rosen PC , Holm P (2017). Long-term changes in abundances of Sonoran Desert lizards reveal complex responses to climatic variation. Global Change Biology 23, 5492–508. 28712135 10.1111/gcb.13813

[inz212396-bib-0029] Flores-Villela O , Canseco-Márquez L (2007). Riqueza de la Herpetofauna. In: Luna I, Morrone JJ, Espinosa D, eds. Biodiversidad de la Faja Volcánica Transmexicana. CONABIO-UNAM, México, pp. 407–20.

[inz212396-bib-0030] Franklin AB (2001). Exploring ecological relationships in survival and estimating rates of population change using program Mark. In: Field R, Warren RJ, Okarma HK, Sievert PR, eds. Wildlife, Land and People: Priorities for the 21st Century. The Wildlife Society, Bethesda, MD, pp. 350–6.

[inz212396-bib-0031] Godínez-Cano E (1985). Ciclo reproductivo de Sceloporus megalepidurus Smith (Reptilia: Sauria: Iguanidae), en la parte oriental de Tlaxcala, México. Thesis, ENEP‐Iztacala, Universidad Nacional Autónoma de México, México.

[inz212396-bib-0032] Gómez Mendoza J (2007). Contribución al conocimiento de la herpetofauna del municipio de Tepeji del Río de Ocampo, Hidalgo. Thesis, FES‐Iztacala, Universidad Nacional Autónoma de México, México.

[inz212396-bib-0033] González-Ruiz GA (1991). Aspectos de la ecología poblacional de Sceloporus megalepidurus Smith (Reptilia; Sauria; Iguanidae), en el oriente de Tlaxcala, México. Thesis, ENEP‐Iztacala, Universidad Nacional Autónoma de México, México.

[inz212396-bib-0034] Grosbois V , Gimenez O , Gaillard J-M *et al* (2008). Assessing the impact of climate variation on survival in vertebrate populations. Biological Reviews 83, 357–99. 18715402 10.1111/j.1469-185X.2008.00047.x

[inz212396-bib-0035] Guillette LJ , Casas-Andreu G (1980). Fall reproductive activity in the high altitude Mexican lizard, Sceloporus grammicus microlepidotus. Journal of Herpetology 14, 143–7.

[inz212396-bib-0036] Harshman LG , Zera AJ (2007). The cost of reproduction: the devil in the details. Trends in Ecology and Evolution 22, 80–6. 17056152 10.1016/j.tree.2006.10.008

[inz212396-bib-0037] Havemann CP , Retief TA , Tosh CA , de Bruyn PJN (2016). Roan antelope *Hippotragus equinus* in Africa: a review of abundance, threats and ecology. Mammal Review 46, 144–58.

[inz212396-bib-0038] Hernández Márquez AE (2016). Ecología térmica y uso de hábitat de una población de Sceloporus megalepidurus (Squamata: Phrynosomatidae) que ocurre en los alrededores de la laguna de Atexcac, Puebla (thesis). FES‐Zaragoza, Universidad Nacional Autónoma de México, México.

[inz212396-bib-0039] Huey RB , Deutsch CA , Tewksbury JJ *et al* (2009). Why tropical forest lizards are vulnerable to climate warming. Proceedings of the Royal Society B: Biological Sciences 276, 1939–48. 10.1098/rspb.2008.1957 PMC2677251 19324762

[inz212396-bib-0040] Jiménez-Cruz E , Ramírez-Bautista A , Marshall JC , Lizana-Avia M , Nieto-Montes de Oca A (2005). Reproductive cycle of *Sceloporus grammicus* (Squamata: Phrynosomatidae) from Teotihuacán, México. The Southwestern Naturalist 50, 178–87.

[inz212396-bib-0041] Jones SM , Ballinger RE (1987). Comparative life histories of *Holbrookia maculata* and *Sceloporus undulatus* in Western Nebraska. Ecology 68, 1828–38. 29357151 10.2307/1939874

[inz212396-bib-0042] Jónsson JE , Gardarsson A , Gill JA , Pétursdóttir UK , Petersen A , Gunnarsson TG (2013). Relationships between long‐term demography and weather in a sub‐arctic population of common eider. PLoS ONE 8, e67093. 23805292 10.1371/journal.pone.0067093 PMC3689676

[inz212396-bib-0043] Katz EM , Tolley KA , Altwegg R (2013). Survival and abundance of Cape dwarf chameleons, *Bradypodion pumilum*, inhabiting a transformed, semi‐urban wetland. Herpetological Journal 23, 179–86.

[inz212396-bib-0044] Kearney MR (2013). Activity restriction and the mechanistic basis for extinctions under climate warming. Ecology Letters 16, 1470–9. 24118740 10.1111/ele.12192

[inz212396-bib-0045] Kearney M , Porter WP (2004). Mapping the fundamental niche: Physiology, climate, and the distribution of a nocturnal lizard. Ecology 85, 3119–31.

[inz212396-bib-0046] Kendall WL (1999). Robustness of closed capture–recapture methods to violations of the closure assumption. Ecology 80, 2517–25.

[inz212396-bib-0047] Kendall WL , Pollock KH , Brownie C (1995). A likelihood‐based approach to capture‐recapture estimation of demographic parameters under the robust design. Biometrics 51, 293–308. 7766783

[inz212396-bib-0048] Kendall WL , Nichols JD , Hines JE (1997). Estimating temporary emigration using capture–recapture data with Pollock's robust design. Ecology 78, 563–78.

[inz212396-bib-0049] LaManna JA , George TL , Saracco JF , Nott MP , DeSante DF (2012). El Niño–Southern Oscillation influences annual survival of a migratory songbird at a regional scale. The Auk 129, 734–43.

[inz212396-bib-0050] Le Galliard JF , Marquis O , Massot M (2010). Cohort variation, climate effects and population dynamics in a short‐lived lizard. Journal of Animal Ecology 79, 1296–307. 20649911 10.1111/j.1365-2656.2010.01732.x

[inz212396-bib-0051] Levy O , Buckley LB , Keitt TH *et al* (2015). Resolving the life cycle alters expected impacts of climate change. Proceedings of the Royal Society B: Biological Sciences 282, 20150837. 10.1098/rspb.2015.0837 PMC4632613 26290072

[inz212396-bib-0052] Leyte-Manrique A , Ramírez-Bautista A (2010). Diet of two populations of *Sceloporus grammicus* (Squamata: Phrynosomatidae) from Hidalgo, Mexico. The Southwestern Naturalist 55, 98–103.

[inz212396-bib-0053] Marler CA , Walsberg G , White ML , Moore M , Marler CA (1995). Increased energy expenditure due to increased territorial defense in male lizards after phenotypic manipulation. Behavioral Ecology and Sociobiology 37, 225–31.

[inz212396-bib-0054] Martínez-Méndez N , Méndez-de la Cruz FR (2007). Molecular phylogeny of the *Sceloporus torquatus* species‐group (Squamata: Phrynosomatidae). Zootaxa 1609, 53–68.

[inz212396-bib-0055] Mayhew WW (1963). Reproduction in the Granite spiny lizard, Sceloporus orcutti. Copeia 1963, 144–52.

[inz212396-bib-0056] Merritt JF , Lima M , Bozinovic F (2001). Seasonal regulation in fluctuating small mammal populations: feedback structure and climate. Oikos 94, 505–14.

[inz212396-bib-0057] McCoy ED , Hartmann PP , Mushinsky HR (2004). Population biology of the rare Florida scrub lizard in fragmented habitat. Herpetologica 60, 54–61.

[inz212396-bib-0058] McKay JL , Phillips BL (2012). Climatic determinants of the reproductive timing in the Asian house gecko, *Hemidactylus frenatus* Duméril and Bibron (Gekkonidae). The Raffles Bulletin of Zoology 60, 583–8.

[inz212396-bib-0059] Niewiarowski PH , Angilletta MJ , Leaché AD (2004). Phylogenetic comparative analysis of life‐history variation among populations of the lizard *Sceloporus undulatus*: An example and prognosis. Evolution 58, 619–33. 15119445 10.1111/j.0014-3820.2004.tb01684.x

[inz212396-bib-0060] Oro D , Torres R , Rodríguez C , Drummond H (2010). Climatic influence on demographic parameters of a tropical seabird varies with age and sex. Ecology 91, 1205–14. 20462134 10.1890/09-0939.1

[inz212396-bib-0061] Ortega A , Barbault R (1984). Reproductive cycles in the Mesquite lizard Sceloporus grammicus. Journal of Herpetology 18, 168–75.

[inz212396-bib-0062] Ortega-León AM , Smith ER , Zúñiga-Vega JJ , Mendez-de la Cruz FR (2007). Growth and demography of one population of the lizard Sceloporus mucronatus mucronatus. Western North American Naturalist 67, 492–502.

[inz212396-bib-0063] Ortega-Rubio A , Halffter G , Barbault R (2000). Bunch grass lizard, *Sceloporus scalaris*, population dynamics at La Michilia Biosphere Reserve, Mexico. Herpetological Journal 10, 33–9.

[inz212396-bib-0064] Owen-Smith N , Mason DR , Ogutu JO (2005). Correlates of survival rates for 10 African ungulate populations: density, rainfall and predation. Journal of Animal Ecology 74, 774–88.

[inz212396-bib-0065] Pastro LA , Dickman CR , Letnic M (2013). Effects of wildfire, rainfall and region on desert lizard assemblages: the importance of multi‐scale processes. Oecologia 173, 603–14. 23494288 10.1007/s00442-013-2642-7

[inz212396-bib-0066] Paxton KL , Cohen EB , Paxton EH , Németh Z , Moore FR (2014). El Niño-Southern Oscillation is linked to decreased energetic condition in long‐distance migrants. PLoS ONE 9, e95383. 24788978 10.1371/journal.pone.0095383 PMC4008376

[inz212396-bib-0067] Pérez-Mendoza HA , Zúñiga-Vega JJ (2014). A test of the fast‐slow continuum model of life‐history variation in the lizard Sceloporus grammicus. Evolutionary Ecology Research 16, 235–48.

[inz212396-bib-0068] Pérez-Mendoza HA , Zúñiga-Vega JJ , Martorell C *et al* (2014). Patterns of spatio‐temporal variation in the survival rates of a viviparous lizard: the interacting effects of sex, reproductive trade‐offs, aridity, and human‐induced disturbance. Population Ecology 56, 605–18.

[inz212396-bib-0069] Pollock KH , Nichols JD , Brownie C , Hines JE (1990). Statistical inference for capture–recapture experiments. Wildlife Monographs 1990, 1–97.

[inz212396-bib-0070] Pradel R (1996). Utilization of capture‐mark-recapture for the study of recruitment and population growth rate. Biometrics 52, 703–9.

[inz212396-bib-0071] Ramírez-Bautista A , González-Romero A (2002). Some reproductive and feeding characteristics of the viviparous Mexican lizard *Sceloporus torquatus* (Phrynosomatidae). The Southwestern Naturalist 47, 98–102.

[inz212396-bib-0072] Ramírez-Bautista A , Jiménez-Cruz E , Marshall JC (2004). Comparative life history for populations of the *Sceloporus grammicus* complex (Squamata: Phrynosomatidae). Western North American Naturalist 64, 175–83.

[inz212396-bib-0073] Ramírez-Bautista A , Ortíz-Cruz AL , Arizmendi MDC , Campos J (2005). Reproductive characteristics of two syntopic lizard species, *Sceloporus gadoviae* and *Sceloporus jalapae* (Squamata: Phrynosomatidae), from Tehuacan Valley, Puebla, Mexico. Western North American Naturalist 65, 202–9.

[inz212396-bib-0074] Ramírez-Bautista A , Hernández-Salinas U , García-Vázquez UO , Leyte-Manrique A , Canseco-Márquez L (2009). Herpetofauna del Valle de México: diversidad y conservación, 1st edn. UAEH-CONABIO, Hidalgo, México.

[inz212396-bib-0075] Ramírez-Bautista A , Stephenson BP , Lozano A , Uribe-Rodríguez H , Leyte Manrique A (2012). Atypical reproductive cycles in a population of *Sceloporus grammicus* (Squamata: Phrynosomatidae) from the Mexican Plateau. Ecology and Evolution 2, 1903–13. 22957191 10.1002/ece3.310 PMC3433993

[inz212396-bib-0076] Ramírez-Bautista A , Hernández-Salinas U , Zamora-Abrego JG (2016). Growth ecology of the tree lizard *Urosaurus bicarinatus* (Squamata: Phrynosomatidae), in a tropical dry forest of the Chamela region, Mexico. Animal Biology 66, 189–99.

[inz212396-bib-0077] Read JL , Kovac K-J , Brook BW , Fordham DA (2012). Booming during a bust: Asynchronous population responses of arid zone lizards to climatic variables. Acta Oecologica 40, 51–61.

[inz212396-bib-0078] Rojo A , Rodríguez J (2002). La flora del Pedregal de San Ángel. 1st edn. INE-SEMARNAT, México.

[inz212396-bib-0079] Roth-Monzón AJ , Mendoza-Hernández AA , Flores-Villela O (2018). Amphibian and reptile biodiversity in the semi‐arid region of the municipality of Nopala de Villagrán, Hidalgo, Mexico. PeerJ 6, e4202. 29312825 10.7717/peerj.4202 PMC5756618

[inz212396-bib-0080] Rzedowski J (2016). Vegetación de México, 1st dig edn. CONABIO, México.

[inz212396-bib-0081] Sánchez-Herrera O (1980). Diagnosis preliminar de la herpetofauna de Tlaxcala, México. Thesis, Facultad de Ciencias, Universidad Nacional Autónoma de México, México.

[inz212396-bib-0082] Sandvik H , Erikstad K , Sæther B-E (2012). Climate affects seabird population dynamics both via reproduction and adult survival. Marine Ecology Progress Series 454, 273–84.

[inz212396-bib-0083] Sillett TS , Holmes RT , Sherry TW (2000). Impacts of a global climate cycle on population dynamics of a migratory songbird. Science 288, 2040–3. 10856216 10.1126/science.288.5473.2040

[inz212396-bib-0084] Sinervo B , Méndez-de la Cruz F , Miles DB *et al* (2010). Erosion of lizard diversity by climate change and altered thermal niches. Science 328, 894–9. 20466932 10.1126/science.1184695

[inz212396-bib-0085] Siqueira CDC , Rocha, CFD (2008). Predation by lizards as a mortality source for juvenile lizards in Brazil. South American Journal of Herpetology 3, 82–7.

[inz212396-bib-0086] Sites JW , Archie JW , Cole CJ , Flores-Villela O (1992). A review of phylogenetic hypotheses for lizards of the genus *Sceloporus* (Phrynosomatidae): Implications for ecological and evolutionary studies. Bulletin of the American Museum of Natural History no. 213.

[inz212396-bib-0087] Smith AL , Bull CM , Driscoll DA (2012). Post-fire succession affects abundance and survival but not detectability in a knob‐tailed gecko. Biological Conservation 145, 139–47.

[inz212396-bib-0088] Smith GR , Lemos-Espinal JA (2005). Comparative escape behavior of four species of Mexican phrynosomatid lizards. Herpetologica 61, 225–32.

[inz212396-bib-0089] Smith HM (1936). Descriptions of new species of lizards of the genus *Sceloporus* from Mexico. Proceedings of the Biological Society of Washington 49, 87–96.

[inz212396-bib-0090] Smith HM (1939). The Mexican and Central American lizards of the genus Sceloporus. Field Museum of Natural History, Publications in Zoology Series 26, 1–397.

[inz212396-bib-0091] Ujvari B , Fisher P , Rydell J , Wahlgren R , Wright B , Madsen T (2015). Population demography of Frillneck lizards *(Chlamydosaurus kingii*, Gray 1825) in the wet‐dry tropics of Australia. Austral Ecology 40, 60–6.

[inz212396-bib-0092] Vercken E , Sinervo B , Clobert J (2012). The importance of a good neighborhood: dispersal decisions in juvenile common lizards are based on social environment. Behavioral Ecology 23, 1059–67.

[inz212396-bib-0093] Vogel P (1984). Seasonal hatchling recruitment and juvenile growth of the lizard Anolis lineatopus. Copeia 1984, 747–57.

[inz212396-bib-0094] Wang G , Hobbs NT , Twombly S *et al* (2009). Density dependence in northern ungulates: interactions with predation and resources. Population Ecology 51, 123–32.

[inz212396-bib-0095] Warner DA , Shine R (2008). Determinants of dispersal distance in free‐ranging juvenile lizards. Ethology 114, 361–8.

[inz212396-bib-0096] Weiss SL (2001). The effect of reproduction on food intake of a sit‐and-wait foraging lizard, Sceloporus virgatus. Herpetologica 57, 138–46.

[inz212396-bib-0097] White GC , Burnham KP (1999). Program MARK: Survival estimation from populations of marked animals. Bird Study 46, S120–39.

[inz212396-bib-0098] Wolf AJ , Hellgren EC , Schauber EM *et al* (2014). Variation in vital‐rate sensitivity between populations of Texas horned lizards. Population Ecology 56, 619–31.

[inz212396-bib-0099] Wright LJ , Hoblyn RA , Green RE *et al* (2009). Importance of climatic and environmental change in the demography of a multi‐brooded passerine, the wood‐lark Lullula arborea. Journal of Animal Ecology 78, 1191–202. 19594660 10.1111/j.1365-2656.2009.01582.x

[inz212396-bib-0100] Zúñiga-Vega JJ (2011). Estimating potential reproductive costs in the survival of a xenosaurid lizard. Herpetological Journal 21, 117–29.

