# Supplementary material for: Population dynamics of three lizard species from the genus Sceloporus: short‐term changes in demographic parameters
Source: Integr Zool. 2019 Nov 25;14(6):542–60. doi: 10.1111/1749-4877.12396 (PMC6899941; doi:10.1111/1749-4877.12396)
Supplement: Supplementary file 1 — Supporting Information [file INZ2-14-542-s001.pdf]

Integrative Zoology 2019; 14: 542–560 doi: 10.1111/1749-4877.12396

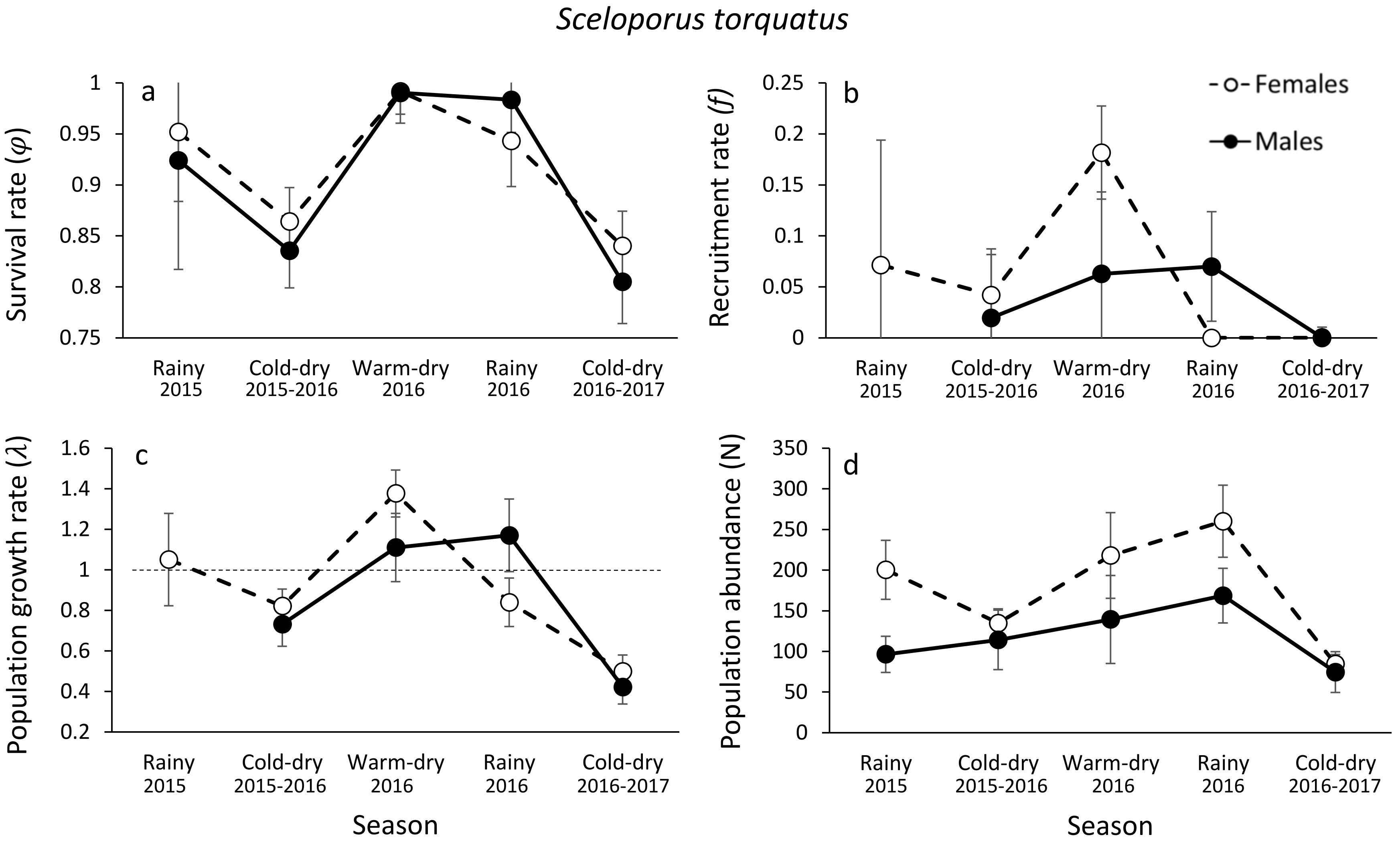

Figure S1 Model-averaged estimates of survival (a), recruitment (b), population growth (c), and population abundance (d) for males and females of *Sceloporus torquatus.* These demographic estimates account for potential differences between years. Error bars indicate one standard error.

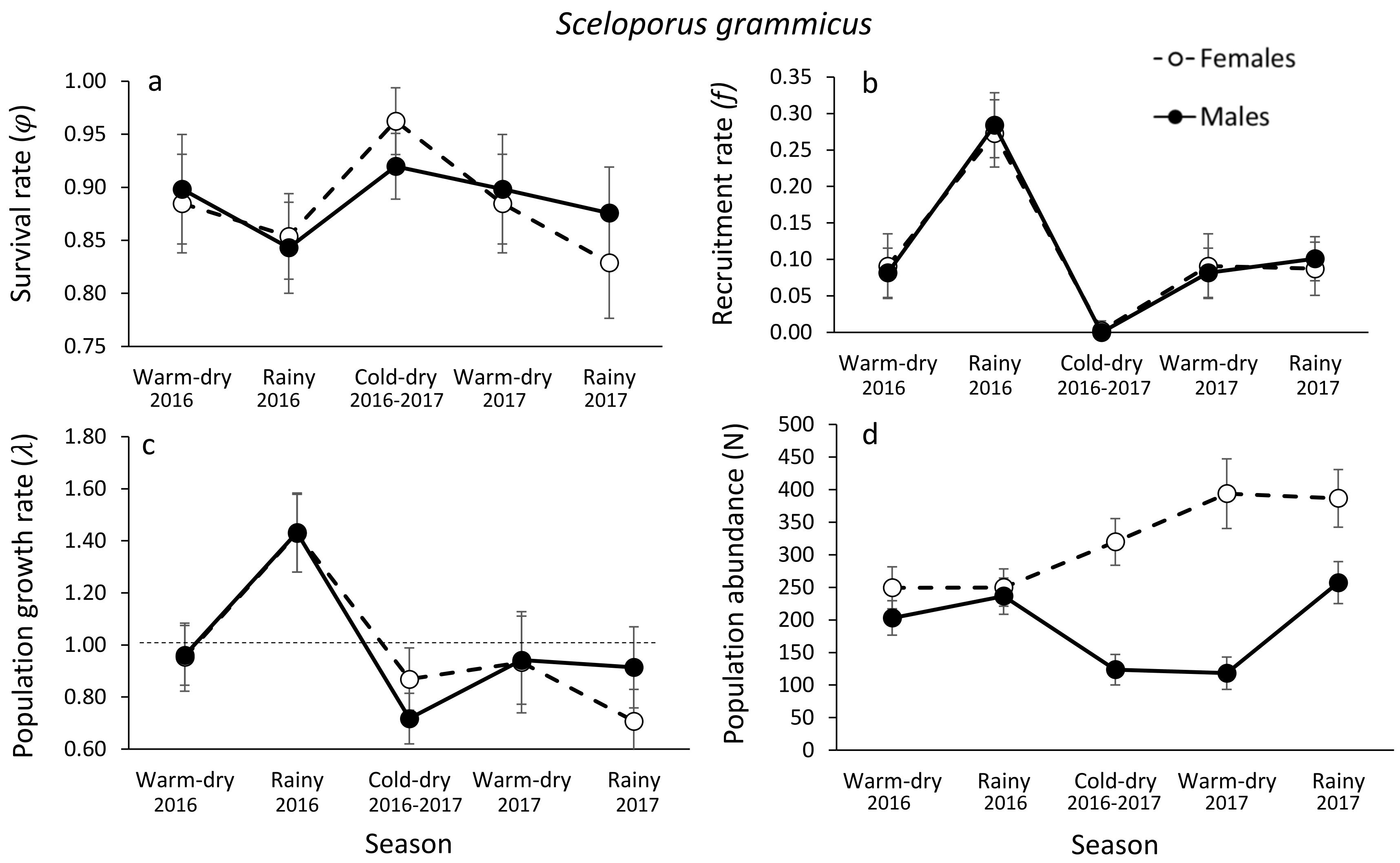

Figure S2 Model-averaged estimates of survival (a), recruitment (b), population growth (c), and population abundance (d) for males and females of *Sceloporus grammicus*. These demographic estimates account for potential differences between years. Error bars indicate one standard error.

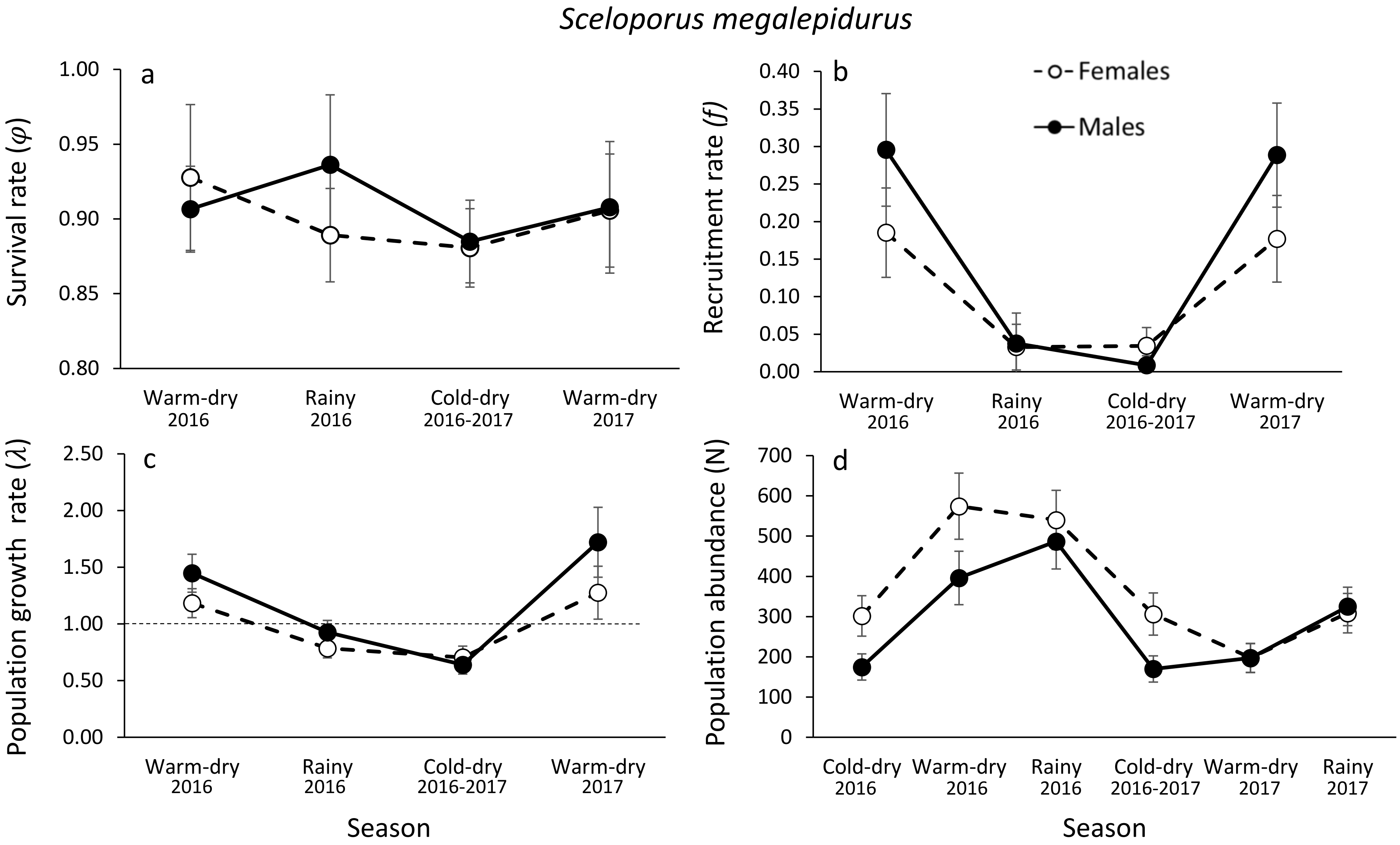

Figure S3 Model-averaged estimates of survival (a), recruitment (b), population growth (c), and population abundance (d) for males and females of *Sceloporus megalepidurus.* These demographic estimates account for potential differences between years. Error bars indicate one standard error.
